# Surface Treatment of Carbon Fibers by Oxy-Fluorination

**DOI:** 10.3390/ma12040565

**Published:** 2019-02-14

**Authors:** Iris Kruppke, Christina Scheffler, Frank Simon, Rolf-Dieter Hund, Chokri Cherif

**Affiliations:** 1 Institute of Textile Machinery and High-Performance Material Technology, Technische Universität Dresden, Hohe Straße 6, 01069 Dresden, Germany; rolf-dieter.hund@tu-dresden.de (R.-D.H.); chokri.cherif@tu-dresden.de (C.C.); 2 Leibniz-Institut für Polymerforschung Dresden e. V. (IPF), Hohe Straße 6, 01069 Dresden, Germany; scheffler@ipfdd.de (C.S.); frsimon@ipfdd.de (F.S.)

**Keywords:** fiber, surface modification, composite materials, microstructures

## Abstract

In this paper, the oxy-fluorination process and the influence of different concentrations of fluorine and oxygen in the gas phase on the physicochemical properties of polyacrylonitrile(PAN)-based carbon fibers are described. The properties of the treated carbon structures are determined by zeta potential and tensiometry measurements. In addition, changes in surface composition and morphology are investigated by X-ray photoelectron spectroscopy (XPS) and scanning electron microscopy (SEM). Adhesion properties are characterized by the single fiber pull-out (SFPO) test. Furthermore, changes in intrinsic properties are described by means of tensile and density measurements. After a primary desizing effect by oxy-fluorination, an increased number of oxygen-containing surface functional groups could be detected, which led to more debonding work in SFPOs with an epoxy-based matrix. It was also shown that the polar surface energy grows with rising fluorine concentration in the reaction gas mixture. In addition, a minor increase of ~10% in the maximum strength of PAN-based carbon fibers is detected by single fiber tensile measurements after oxy-fluorination with a fluorine content of 5% in the reaction mixture.

## 1. Introduction

None of the great advantages of carbon fibers as reinforcing material for different composite types, including their high specific stiffness or strength, can come into effect without surface functionalization. Therefore, the structural application potential of fiber-reinforced polymers is limited by component properties prior to their processing and service in the aerospace or automotive industries or external loading, such as impact or tensile stress [1]. Over the last decades, various methods have been developed and implemented in order to achieve good adhesion properties between this highly inert material and sizings or matrix materials [2,3]. Wet-chemical and electrochemical methods of oxidizing the fiber surface prior to sizing are the state of the art in carbon fiber production. Here, layers near the outer surface are removed to avoid weak boundary layers, which decrease adhesion properties. Oxidized surface functional groups as well as a modified surface roughness are introduced to ensure a form-fit connection or under-cut geometrical features. Typically, different acids are used for these treatments, such as sulfuric acid [4,5], aqueous potassium nitrate [6,7], methanesulfonic acid [8], nitric acid [9,10,11], or phosphoric acid [12]. Additionally, several atmospheric plasma technologies based on various process gases are nowadays commonly used to produce functional groups on a carbon fiber surface. The gases range from non-reactive gases, such as nitrogen, argon, carbon dioxide, or tetrafluoromethane, to reactive gases, such as oxygen, technical air, nitrogen dioxide, and nitric oxide [9,13,14,15,16,17,18,19,20,21,22].

As novel fields of application for fiber-reinforced composites are constantly emerging, requirements imposed on sizings and surface modifications become more specific according to their implementation. Hence, alternative methods have come into focus, such as fluorination [23,24,25] and oxy-fluorination treatments resulting in a high functionalized surface of polyacrylonitrile (PAN)-based carbon fibers. Previously, oxy-fluorination had only been used for initial studies, as it was not suitable for treatments on a production scale. Fluorination and oxy-fluorination methods are exclusively described for polymeric materials by Kharitonov et al. [26,27,28,29,30]. The main aspects of their research involved diffusion barriers by fluorination as well as surface activation by oxy-fluorination. In terms of carbon fibers, initial investigations on the intercalation of fluorine in PAN-based carbon fibers were completed using fluorination by Mathur et al. [23]. Their work revealed the effects of the intercalation of fluorine as a heteroatom on the stiffness and Young’s modulus, suggesting improved mechanical properties. X-ray photoelectron spectroscopy (XPS) studies by Tressaud et al. investigated the effect of fluorine on different carbon fibers, for example, based on PAN or pitch [25]. As a result, a dependence on the degree of graphitization of the fibers used could be shown. The higher the graphitization, the higher was the amount of intercalated fluorine, which was accompanied by a change from ionic to covalent bonding between fluorine and carbon. Park et al. used an oxy-fluorination procedure at higher temperatures that led to an increase in both the investigated interlaminar shear stress (ILSS) and the surface polarity of the fibers [1]. In that case, an oxy-fluorination procedure was used to functionalize PAN-based carbon fibers, whereby improved adhesion could be demonstrated. Moreover, increased surface free energy and enhanced adhesion to thermoplastic polymers after oxy-fluorination were demonstrated by Käppler et al. [31]. This increase in surface functionality was proven for other types of high-performance fibers as well [32].

The research described in this paper reveals the effects of different concentrations of fluorine and oxygen in the gas phase during the modification process of carbon fibers. For the first time, a production-scale batch reactor was available to investigate the influence of oxy-fluorination on carbon fibers and to verify several suggestions previously expressed in the literature [23,24,25]. As a reference material, polyurethane-sized PAN-based carbon fibers were used and compared to oxy-fluorinated and fluorinated fibers. Different surface-sensitive methods and microscopy were used to describe the changed surface composition using electrokinetic measurements, tensiometry, Raman spectroscopy, and XPS. In accordance with the technical field of application of carbon fibers, textile physical properties and bond strength to the matrix were determined by means of tensile measurements and single fiber pull-out tests. The achieved results exhibited an improved surface composition after the oxy-fluorination treatment, leading to the assumption that this surface treatment is a very promising surface functionalization method prior to sizing as part of the production process of carbon fibers due to their missing wet-chemical nature. The need for electrolyte baths or acids, as well as a reconditioning of process media, could be avoided by the use of a continuous oxy-fluorination plant. In addition, oxy-fluorination offers the advantage of affecting the mechanical performance of treated carbon fibers. Based on the introduced method, the intrinsic properties and internal structure of carbon fibers can be influenced by adding a process step to the standard procedure [33] with specific manufacturing process conditions during stabilization, carbonization, and graphitization, or precursor composition.

## 2. Materials and Methods

### 2.1. Materials

The commercially available PAN-based carbon fiber HTS 40 F13 was purchased from Teijin Carbon Europe GmbH (Wuppertal, Germany). It was endowed with an epoxy/polyurethane sizing. The fineness was 800 tex, 12 K, and the single fiber diameter was 7 μm on average. For comparative purposes, natural graphite with a high purity and a particle size defined by a 50 mesh was provided. The ash content amounted to < 0.15%.

For the single fiber pull-out test, a thermosetting matrix consisting of Epikote^TM^ resin MGS^®^ RIMR 135 from Hexion (Momentive^TM^, Columbus, GA, USA) based on bisphenol A [4,4′-(propane-2,2-diyl)diphenol] resin and an Epicure^TM^ Curing Agent MGS^®^ RIMH 137 based on alkyl ether amine and isophorone diamine [3-(aminomethyl)-3,5,5-trimethylcyclohexan-1-amine] (Momentive^TM^, Columbus, GA, USA) wasemployed.

### 2.2. Scanning Electron Microscopy

The selected carbon fibers were investigated by scanning electron microscopy (SEM) prior to and after oxy-fluorination to study changes in surface morphology. To characterize the treated samples, carbon fibers were placed on a specimen holder with a carbon pad to ensure conductibility without any further treatment for surface functionalization. Here, a Quanta 250 FEG ESEM™ from Thermo Fisher Scientific (Waltham, MA, USA) was used at 14.00 kV and 200 Pa. Fiber surface studies before and after single fiber pull-out were performed by scanning electron microscopy using the SE2 detector of an ULTRA PLUS microscope (Carl Zeiss Microscopy GmbH, Jena, Germany).

### 2.3. X-ray Photoelectron Spectroscopy

All XPS studies were carried out by means of an Axis Ultra photoelectron spectrometer (Kratos Analytical, Manchester, UK). This spectrometer was equipped with a monochromatic Al Kα (hν = 1486.6 eV) X-ray source of 300 W at 15 kV. The kinetic energy of photoelectrons was determined with a hemispheric analyzer set to pass energy of 160 eV for wide-scan spectra and 20 eV for high-resolution spectra. The carbon fiber sample was mounted on a sample holder with adhesive tape so that the area to be analyzed was positioned over a hole in the sample holder. Although the carbon fibers were electrically conductive, a low-energy electron source working in combination with a magnetic immersion lens was employed to avoid any electrostatic charging of the sample that might be caused by fixing the fibers on the sample holder with the insolating adhesive tape. Later, all recorded peaks were shifted by the same value that was necessary to set the C 1s component peak of saturated hydrocarbons to 285.00 eV. The maximum information depth of the XPS method is about 8 nm.

### 2.4. Electrokinetic Measurements

To study the changed surface functionalities of carbon fibers and their behavior in contact with aqueous solutions, streaming potential measurements were carried out to determine the electrokinetic potential (zeta-potential, ζ). Based on a fiber cell containing about 1 g of carbon fiber samples, streaming potential measurements were performed by a SurPASS 3 electrokinetic analyzer (EKA) from Anton Paar (Graz, Austria). The streaming potential measurements were carried out across a range pH values of a 1 × 10^−3^ mol·L^−1^ aqueous KCl solution. The pH values were adjusted with 0.1 mol·L^−1^ of HCl or KOH.

### 2.5. Single Fiber Tensile Test

Since changes in the surface structure are known to affect mechanical fiber properties, single fiber tensile tests were used to evaluate the fiber strength of untreated as well as treated carbon fibers using a Favimat^+^ from Textechno H. Stein GmbH (Mönchengladbach, Germany). The testing rate was defined as 10 mm·min^−1^, with having a measuring head of 210 cN. At least 100 single measurements with a clamping length of 10 mm were performed. To evaluate the mechanical properties, a conventional Weibull distribution analysis was carried out.

### 2.6. Determination of the Surface Free Energy

To characterize the wetting behavior, a K100SF tensiometer from Krüss GmbH (Hamburg, Germany) was employed for single fiber tensiometry. The single fibers were separated from the fiber bundle and immersed in distilled water (surface tension at 23 °C, σ^total^ = 72.8 mN·m^−1^) and diiodomethane (> 99%, Sigma-Aldrich Chemie GmbH, surface tension at 23 °C, σ^total^ = 50.8 mN·m^−1^). The measurement was repeated at least ten times for each test liquid. For the measuring procedure, each single fiber was placed on a sample holder, which was connected to a microbalance (sensitivity of ± 0.1 µg) and subsequently immersed into the test liquid (at a depth of 2 mm). The surface free energy determination was carried out according to Owens, Wendt, Rabel, and Kaelble [34,35,36] (Equation (1)).

(1) $$\frac{(1+\cos\theta )\cdot \sigma_{l}}{2\cdot \sqrt{\sigma_{l}^{D}}}=\sqrt{\sigma_{S}^{P}}\cdot \sqrt{\frac{\sigma_{l}^{P}}{\sigma_{l}^{D}}}$$

Here, the total surface free energy *σ^total^* can be calculated by means of the determined contact angles (*θ*) to analyze the contributions of the polar (*σ^P^*) and disperse (*σ^D^*) terms of the surface free energy. The indices *l* and *s* represent the liquid and solid phases, i.e., test liquid and fiber surface, respectively.

### 2.7. Determination of Density

The densities of the surface-treated carbon fibers were determined by a pycnometer according to Gay Lussac made of borosilicate glass 3.3 with a volume of 10 mL (Carl Roth GmbH + Co KG, Karlsruhe Germany). Water was used as a test liquid to measure the fibers’ volumes. For each measurement, 0.5–0.7 g of fibers were employed. For the given mean density values, ten single measurement cycles were averaged.

### 2.8. Oxy-Fluorination Procedure

**Figure 1:**
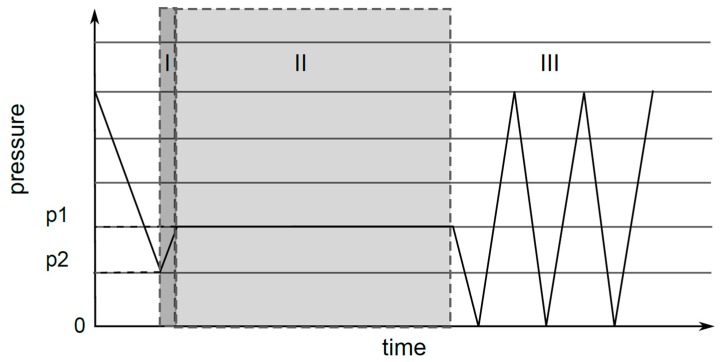
Oxy-fluorination process with evacuation of batch reactor until pressure p2, purging with F_2_/N_2_ mixture (10:90 vol%) until p1 (I), processing (II), and multiple evacuations and purging with air until all remaining fluorine is removed (III).

The oxy-fluorination procedure was carried out as a batch process with carbon fibers fixed to a metal frame. The frame had a dimension of 1.60 m × 0.55 m and allowed the fluorination of about 50 m of yarn per batch process. The modification and subsequent characterization took place with fully elongated fibers excluding the winding section (1.5 m each). For the treatment, different amounts of fluorine (F_2_/N_2_-mixture with 10:90 vol%) were used to functionalize the fibers at room temperature with a reaction time of 180 s (Figure 1).

For the process shown in Figure 1, the reaction chamber was evacuated to the base pressure p_2_. The participating oxygen content results from remaining air that is defined by p2. Therefore, the remaining oxygen in the air corresponded to the amount of oxygen, which was used for oxy-fluorination. Then, the F_2_/N_2_ mixture was added to achieve pressure p1, which was the maintaining pressure over the reaction time. The resulting concentrations of oxygen and fluorine are shown in Table 1. The inward flow of F_2_/N_2_ defined the amount of fluorine used for the reaction (Figure 1I). After completion of the process (Figure 1II), the reaction chamber was evacuated and purged with air several times to completely remove fluorine (Figure 1III). Leftover reaction gas was removed by a CaCO_3_ absorber.

### 2.9. Single Fiber Pull-Out Test

**Figure 2:**
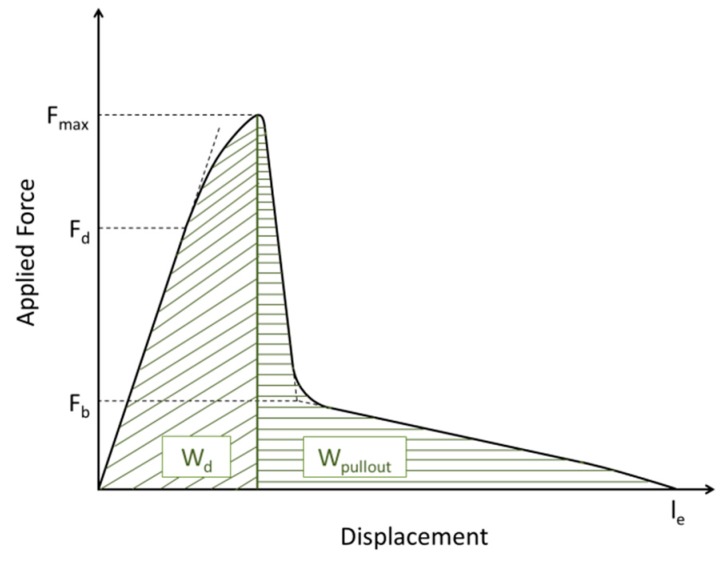
Schematic force-displacement curve recorded during a pull-out test revealing the characteristic points for the calculation of interfacial parameters.

The interfacial adhesion strength was evaluated by a single fiber pull-out test based on embedding equipment designed and constructed by the Leibniz Institute for Polymer Research [37,38]. Model microcomposites were prepared by the computer-controlled embedding of one end of the single fiber into the matrix. It was integrated perpendicularly with a pre-selected embedding length l_e_ (l_e_ = 100 μm) at a controlled atmosphere and temperature. After embedding the fiber at 45 °C, the samples were cured for 1 h at 85 °C and 6 h at 80 °C before being cooled down to ambient temperature. The pull-out test was carried out on a self-built pull-out apparatus with a force accuracy of 1 mN, displacement accuracy of 0.07 μm, and loading rate of 0.01 μm/s in ambient conditions. The force-displacement curves were recorded, and the maximum force (F_max_) required to pull fibers out of the matrix was determined. Subsequent to the testing procedure, the fiber diameter d_f_ was measured by optical microscopy; l_e_ was determined by the force-displacement curve and cross-checked by SEM. The adhesion bond strength between fiber and matrix was characterized by the values of apparent interfacial shear strength (τ_app_ = F_max_·[π·d_f_·l_e_]^−1^); local interfacial shear strength τ_d_, and interfacial frictional stress τ_f_ were calculated based on debonding force F_d_ and frictional force F_b_ [39,40,41] (Figure 2), respectively. The debonding work W_d_ is defined as the area under the curve from l = 0 µm to l at F_max_; the work to pull out a fiber after debonding W_pullout_ ranges from l at F_max_ to the full embedding length l_e_, where the force F reaches zero and the fiber is completely pulled out of the matrix. The total pull-out work W_total_ is defined as W_total_ = W_d_ + W_pullout_. Each fiber-matrix combination was evaluated in approximately 15–20 individual tests.

## 3. Results and Discussion

### 3.1. Surface Characterization and Bulk Properties

**Figure 3:**
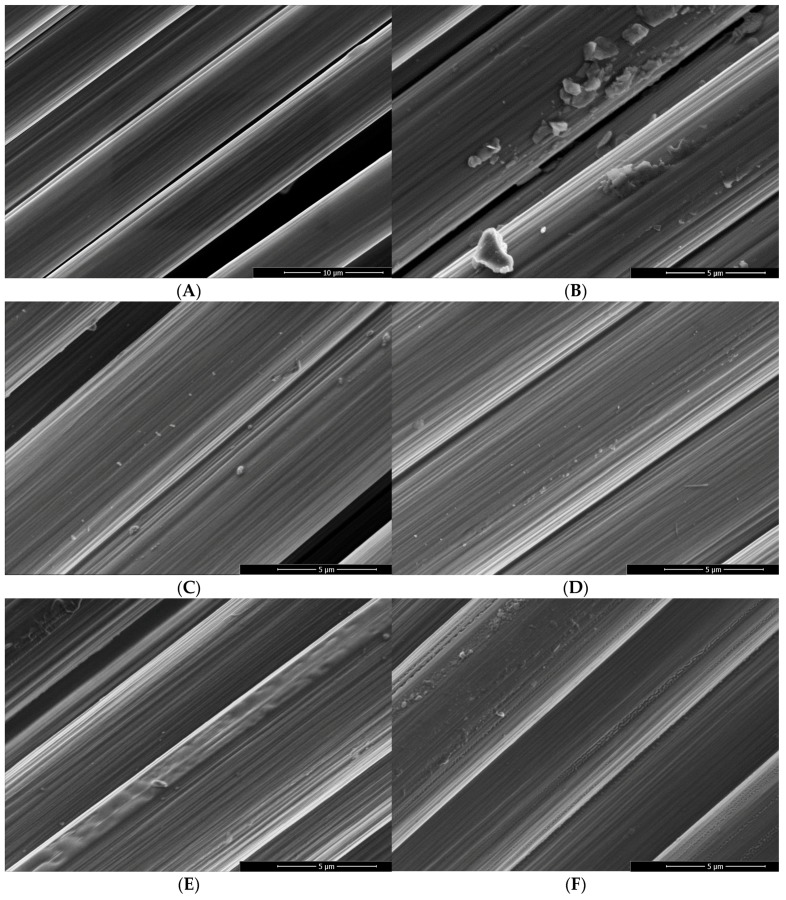
Scanning electron micrographs taken from the untreated reference fiber sample (**A**) and differently oxy-fluorinated carbon fiber samples: 1 vol% fluorine (**B**), 3 vol% fluorine (**C**), 5 vol% fluorine (**D**), 7 vol% fluorine (**E**), and 10 vol% fluorine (**F**).

Oxy-fluorination and fluorination lead to changes in surface morphology, as shown in Figure 3. As can be seen in Figure 3B–F, by increasing the fluorine content in the reaction chamber, the striation that is caused by the stretching of fibers appeared to be more pronounced. This is associated with a roughening of the fibrillary surface structure, which can be considered as result of the oxidation of PAN-based carbon fibers [42]. Here, all modified fibers had porous areas along their axis and exhibited particle contaminations on their surfaces. Figure 3E,F indicated the presence of a porous near-surface layer covering the entire fiber. These findings correspond to previous studies [32]. Comparing the modified fibers to the untreated fiber sample (Figure 3A), the desizing of fibers was observed. During their treatment with high concentration of fluorine in the process gas (10 vol% fluorine), the fibers were completely desized (Figure 3F).

**Figure 4:**
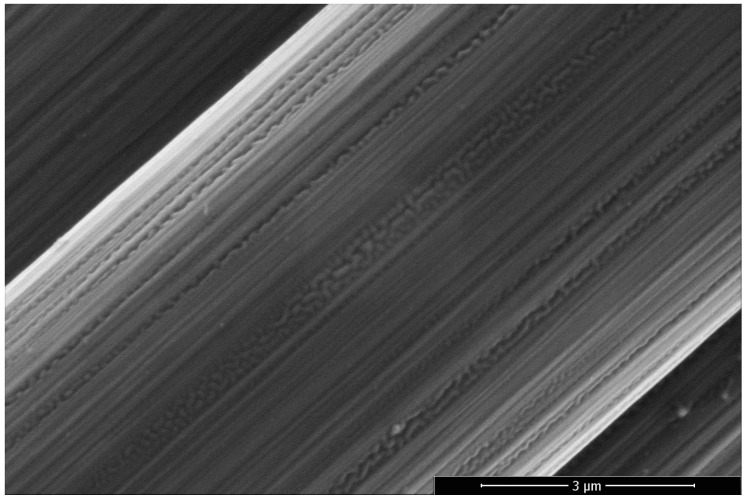
Scanning electron micrographs taken of the 10 vol% fluorine treated sample.

**Figure 5:**
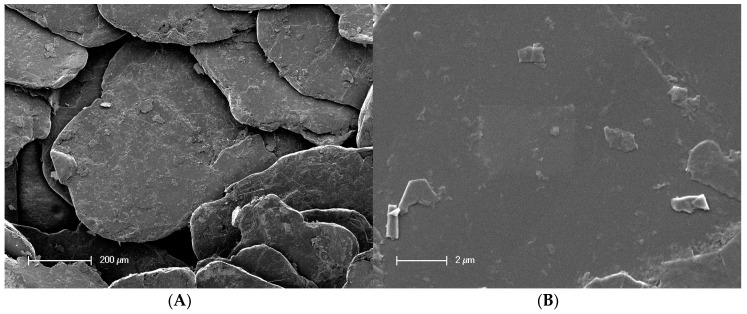

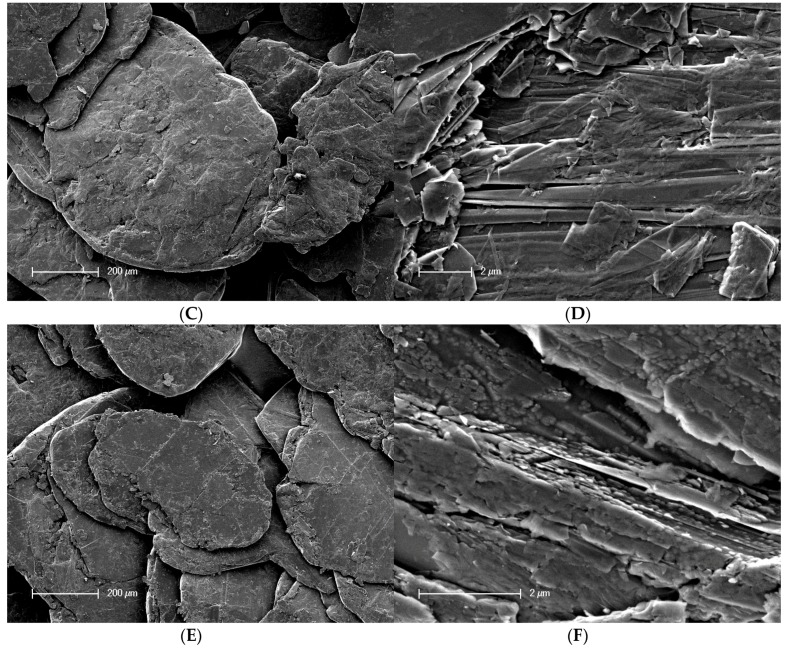
Scanning electron micrographs taken of the untreated reference fiber sample (**A**,**B**) and differently oxy-fluorinated carbon fiber samples: 5 vol% fluorine (**C**,**D**) and 10 vol% fluorine (**E**,**F**).

Moreover, a degradation of the surface in the case of 10 vol% fluorine can be seen in Figure 4. Along the fiber striations and axis, an explicit change in surface morphology and newly formed pores are visible. This behavior was also observed for treated graphite (Figure 5). Compared to the reference sample, the oxidative oxy-fluorination treatment with 5 vol% fluorine led to a roughening of the surface, accompanied by a degradation of the layers beginning from the edge. The fluorination with 10 vol% intensifies the described effects. Especially the degradation and serration along the graphitic layer has to be highlighted, which occurs similarly to the carbon fiber at 10 vol% (cf. Figure 4). Caused by these obvious changes in carbon structure, a change in carbon hybridization for the outer carbon fiber layers is anticipated

**Figure 6:**
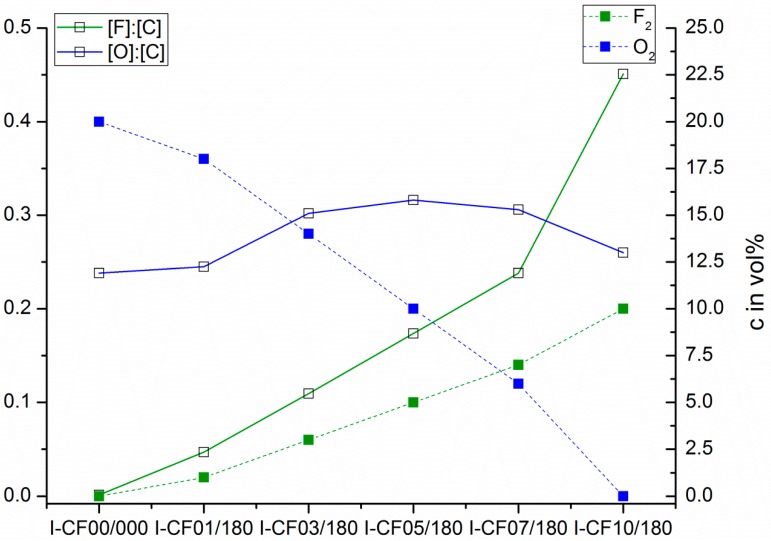
Relative amounts of oxygen ([O]:[C]) and fluorine ([F]:[C]) in dependence on the fluorine content in the process gas used to modify carbon fiber samples.

As shown in Table 2, the mean densities of treated carbon fiber samples can be assumed to be constant with rising fluorine concentrations. Exclusively in the case of 10 vol% fluorine in the gas mixture compared to the reference specimen, a significant difference was found by an analysis of variance with α = 0.05. This is probably caused by the was observed associated fluorine after the treatment, as shown in Figure 6. An increase in density was found by Nanse et al. [43] who attributed this change to an introduced fluorinated monolayer on the graphite-like lattice. Additionally, Mathur et al. [23] analyzed an increase in mass for fluorinated PAN-based carbon fibers by 16% compared to the reference. For our sample series in its absolute values, a maximum increase in density of only 4.9% between the reference and I-CF10/180 was observed.

**Figure 7:**
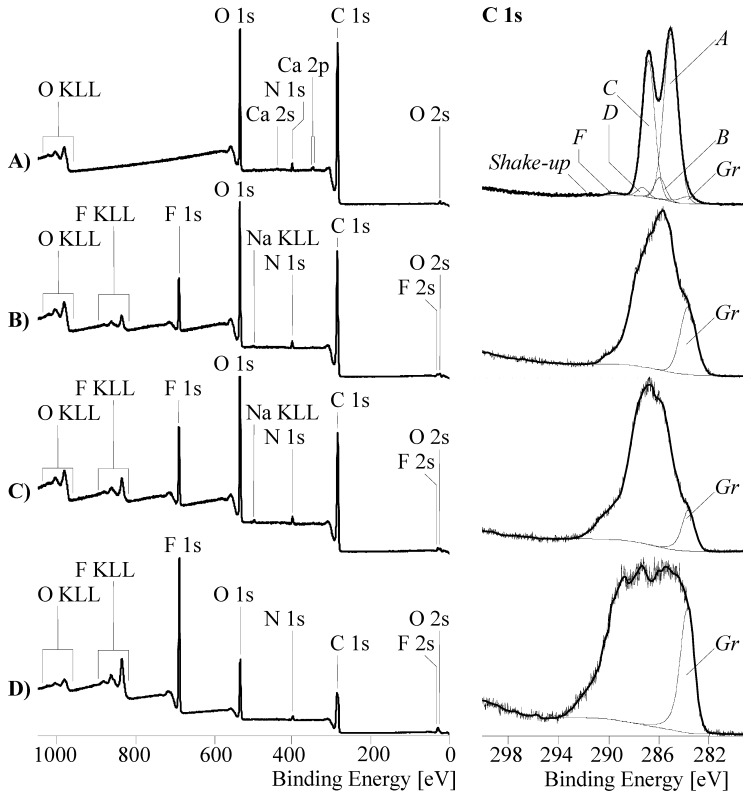
Wide scan (left column) and high-resolution C 1s (right column) XPS spectra recorded from an untreated PAN-based carbon fiber sample (I-CF00/000–A) and carbon fiber samples, which were oxy-fluorinated with 1 vol% (I-CF01/180–B), 5 vol% (I-CF03/180–C), and 10 vol% (I-CF10/180–D) fluorine in the process gas.

XPS spectra recorded from the oxy-fluorinated fiber carbon samples clearly showed the incorporation of considerable amounts of fluorine in the outer carbon fiber surfaces. The relative fluorine content ([F]:[C]) of the carbon fibers increased with the fluorine concentration of the process gas employed for the modification (Figure 6). In contrast, the relative amount of oxygen ([O]:[C]) increased slightly with increasing fluorine content in the process gas. At 5 vol%, the maximum of oxygen-carrying functional groups was observed. As the fluorine content in the process gas was increased further, the number of oxygen-carrying groups decreased in favor of fluorine-containing groups (Figure 6). It can be concluded that with an increased supply of fluorine in the process gas, the initially present or formed oxygen-containing functional groups were further oxidized to small molecules. The free surface sites that have become vacant were immediately oxidized by fluorine to form fluorine-carrying functional groups. These findings appear interesting as they support the assumption that during intensive surface treatments—in contrast to plasma or flame treatments—no weak-bounded layers were formed. The majority of the numerous functional groups were covalently bonded to the carbon fiber surface. This can be illustrated by high-resolution C 1s spectra recorded from the differently treated carbon fibers samples (Figure 7). According to the shape of the C 1s spectrum (Figure 7A) recorded from the reference sample I-CF00/000, the carbon fiber was wrapped with an epoxy network.

The spectrum was deconvoluted into six component peaks showing the different binding states of the carbon atoms (Figure 7). The main component peaks *A* at 285.00 eV as a result from saturated hydrocarbons [44]. The second intense component peak *C* at 286.67 eV shows the presence of C–O ether and alcohol groups, which were formed by opening the oxirane groups during the curing of the epoxide. The C–O bonds were also constituents of the urethane groups in the polyurethane film former. A minor amount of intact oxirane rings was detected as component peak *D* at 287.34 eV. The appearance of the component peaks *B* (at 285.77 eV) and *F* (at 289.5 eV) is based on the presence of the polyurethane film former in the coating layer. Photoelectrons of the amine-sited carbon atoms in the urethane groups contributed to component peak *B*, and the photoelectrons of the corresponding carbonyl carbon atoms contributed to component peak *F*. Since the intensity of component peak *F* was less than the intensity of component peak *B,* C–N bonds of preferably secondary and tertiary amino groups must be present in the coating network. In order to fit the sum curve of all component peaks to the recorded spectrum, it was necessary to introduce a further component peak *Gr* at 283.62 eV [25]. This component peak resulted from carbon atoms in the sp^2^ hybrid state forming the graphite-like lattice of the carbon fibers. Electron transitions between occupied π and unoccupied π* orbitals (π → π*) led to *shake-up* peaks, which were observed at 291.45 eV. The oxidation reactions during the oxy-fluorination process were characterized by the production of oxygen- and fluorine-carrying functional groups (Figure 6). A variety of these groups remain covalently bonded on the carbon fiber surfaces. Due to the newly formed functional groups, the shape of the C 1s spectra changed significantly (Figure 7B–D) [43]. By increasing the amount of fluorine in the gas mixture (which correlated with the degree of surface functionalization), they became wider. Similar spectra were recorded by Sherwood et al. [45] for highly oxidized carbon fibers. The reason for the apparent widening of the C 1s spectra was an increase in the number and intensities of the component peaks resulting from the different functional groups. In fact, it was not possible to unambiguously decompose the C 1s spectra into their component peaks and assign the chemical environment of carbon to each of the individual component peaks. However, based on the changed shape of C 1s spectra, it was concluded that the chemical structure of the sizing network that originally wrapped the carbon fiber was destroyed during the oxy-fluorination (the carbon fibers were desized, which can also be seen in Figure 3).

Furthermore, with an increasing fluorine content in the process gas, the peak flank on the low-energy side of the C 1s spectra appeared to be steeper, which indicated an increase in sp^2^-hybridized carbons [25]. These findings, which can be considered as an uncovering of the graphite-like lattice of the carbon fiber surface, is supported by the oxidative degradation of the sizing discussed above. However, the high number of functional groups that can be recognized based on the high intensities of the C 1s spectra and the binding of energy values higher than 285 eV indicated that the uncovered carbon surface was oxidatively attacked by fluorine.

**Figure 8:**
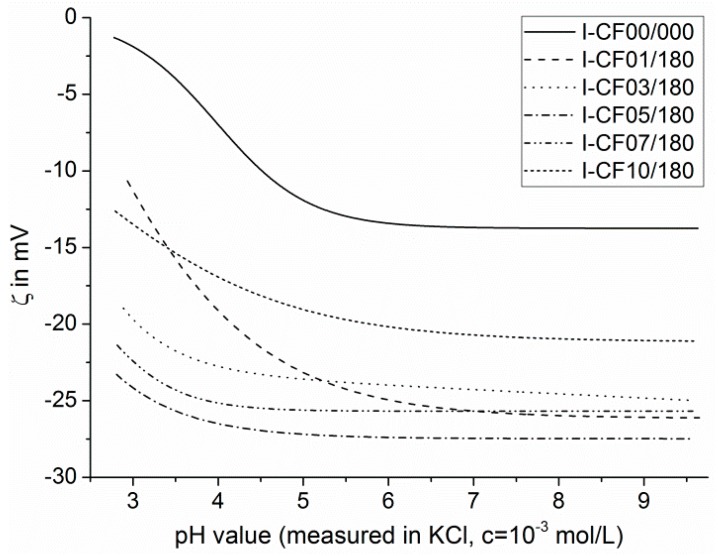
Dependence of the zeta potential (ζ) of differently treated carbon fiber samples on the pH values of a 1 × 10^−3^ mol·L^−1^ aqueous KCl solution.

The desizing of the carbon fibers and their oxidation also affected the interactions between carbon fiber surfaces and probe molecules with which they were contacted in tensiometry (Table 3) and electrokinetic experiments (Figure 8). Both of these processes on the fiber surfaces can be recognized by changes in the total surface free energy (Table 3). Due to the numerous C–O bonds in the sizing covering the carbon fiber, the surface of the sample I-CF00/000 was hydrophilic and can described as polar. As mentioned above, the oxy-fluorination desized the carbon fibers (Table 3). The uncovering of the graphite surface and the production of (per)fluorinated oxidation products (carrying of C–F bonds) [46] increased the water contact angles (*θ_water_*), which corresponded to an decrease in the samples’ surface hydrophilicity and polarity. However, the modified carbon fiber sample surfaces remained spontaneously wettable with water, and the values of the total surface free energy can be–independent of the fluorine concentration in the process gas–considered as moderately polar. This is due to the presence of oxygen-containing functional groups, which may be degradation products of the sizing or may be newly formed by the oxy-fluorination process. The lowest value of the total surface free energy was found for the sample that was treated with a process gas containing 10 vol% fluorine. Figure 6 reveals that the treatment introduced an extraordinarily high number of fluorine-carrying surface groups while the number of oxygen functionalities became minimal.

Since the XPS method provided little insight into the nature and reactivity of the chemically active surface groups, electrokinetic measurements were performed. Figure 8 shows the electrokinetic potential (zeta-potential, ζ) in dependence on the pH value of a 1 × 10^−3^ mol·L^−1^ aqueous KCl solution. During variation of the pH value, the potassium and chlorine ions kept the ionic strength of the aqueous solution nearly constant. XPS studies showed that the surfaces of the sized carbon fibers (I-CF00/000) were not endowed with numerous surface groups able to undergo dissociation reactions in aqueous media. Nevertheless, the shape of the ζ = ζ(pH) curve recorded from sample I-CF00/000 was characteristic for hydrophilic solids. Obviously, negatively charged OH^−^ ions that were specifically adsorbed on the fiber surface led to negative surface potential values, which were observed as negative zeta potential values. Based on an increase in pH, the availability of OH^−^ ions driving the adsorption grows as well. Hence, the zeta potential increased with pH to a maximum value of about 12 mV at pH > 6 (Figure 8). The observed zeta potential maximum corresponded with a constant number of charge carriers on the fiber surface. Thus, at pH > 6 all surface sites were occupied by OH^−^ ions. Due to the hydrophilic surface character of the sized carbon fibers, the OH^−^ ions were not forced to strip off their hydration shells to make space for the adsorption of further OH^−^ ions. Since ether groups were not suitable to be protonated in medium acidic environment, positive zeta potential values were not observed. However, in the acidic range (3 ≤ pH < 4.5), the ζ = ζ(pH) curve seemed to be s-shaped (Figure 8), indicating that a few positively charged amino groups (–N^+^R_2_H) partly compensated the negative surface potential, which was caused by the adsorption of OH^−^ ions.

The decomposition of the sizing and the uncovering of the fiber surfaces during the oxy-fluorination changed the shapes of the ζ = ζ(pH) curves. At a small degree of fluorination (samples I-CF01/180 and I-CF03/180), the zeta potential values did not remain constant at pH > 6. The slight slope resulted from the increased hydrophobicity of the treated carbon fiber surfaces (cf. comparison of water contact angle values, *θ_water_* in Table 3). In order to be stably adsorbed on hydrophobic surface sites, the OH^−^ ions removed their hydration shells so that further OH^−^ ions can be adsorbed and contribute to an increase in charge density on the surface. Due to the maximum content of oxygen-carrying functional surface groups, sample I-CF05/180 was slightly more hydrophilic as the other oxy-fluorinated samples. Hence, a plateau phase of zeta potential values at pH > 6 was observed. Presumably, this plateau can be attributed to the endowing of the fiber surfaces with dissociable groups, such as carboxylic acids. In addition to the XPS studies, this assumption was supported by the lack of an s-shaped curved section at pH < 4.5. Instead, the ζ = ζ(pH) curves of the oxy-fluorinated samples sharply dropped towards zero. Since the isoelectric points (pH values where zeta potential value equals zero, pH|_ζ = 0_) were smaller than three, these characteristic values could not be determined in the electrokinetic experiment. The oxy-fluorination experiment with 10 vol% of fluorine in the process gas resulted in a high fluorine concentration on the fiber surface. As can be seen in Table 3, the fluorine-containing surface groups made the fiber surface of sample I-CF10/180 more hydrophobic, which corresponds to the constant slope of the ζ = ζ(pH) curve at pH > 6.

The desizing effect during oxy-fluorination was confirmed as the carbon content determination by the 1M KCl solutions before and after zeta potential measurements exhibit a high value for the reference sample I-CF00/000. This corresponds to its sized character, which was additionally demonstrated by XPS measurements. With increasing treatment intensity and fluorine content, respectively, the carbon content of the measurement solution decreased to 10% fluorine in the reaction gas mixture. This desizing effect could be observed on the CF surface as well by means of SEM (Figure 3).

### 3.2. Mechanical tests

The oxy-fluorination of carbon fibers results in an increase in maximum fiber tensile stress as listed in Table 4. According to Figure 3, the oxidation of carbon fiber results in a degradation, starting from the surface. Here, also a decrease of density can be expected. With respect to the density measurements (Table 2) and the evaluated titer, this is probably an effect caused by a decrease in diameter of the single fiber. Here, the calculated diameter decreased by approximately 2.1% for a treatment with 1 vol% and by approximately 4.8% for 4.25% by oxy-fluorination with 5 vol% and 10 vol%, respectively. The highest diameter decrease was obtained for the most intense condition at 5 vol%, which corresponds to the findings of the surface characterization in Section 3.1. Considering the theory of brittle materials, the resulting strength is increasing with a reduction in volume, which could be demonstrated by reducing the diameter [47]. In this regard, the intercalation of fluorine into a graphitic structure of PAN-based carbon fibers cannot be exclusively responsible for an increase in tensile strength as shown by Mathur et al. [23]. Compared to the reference fiber, the maximum force rises by approximately 10.1% for the specimen treated with 5% fluorine in the gas mixture. For the stress-strain curves, a slight lag in the beginning was observed, which is related to unidentified mechanisms in the fiber that potentially occur due to its changed surface-near morphology (Figure 4). The results of the stress-strain curves exhibit a unimodal Weibull appearance.

To describe the distribution of the carbon fiber strength after the surface functionalization, a conventional Weibull analysis with a strength distribution was performed. The data were plotted according to Weibull coordinates by Equation (2) [47], where σ is the tensile strength in GPa (fracture stress at the gauge length 10 mm). The influence of the gauge length can be neglected as a short length of 10 mm was used. Furthermore, the probability of failure *P* is given by *(3)* with *i* for sample number and *n* as total number of samples [33,47,48]. For the calculation of considered σ, the measured density of the differently treated carbon fibers was taken from Table 1.

(2) ln(−ln(1−P(σ)))=f(ln(σ))

(3) P=i/(n+1)

**Figure 9:**
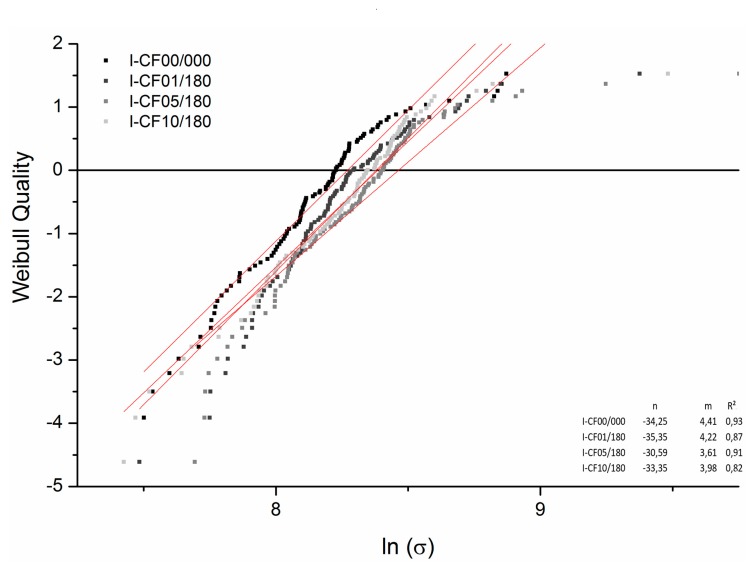
Strength distributions as Weibull plot for I-CF00/000 (reference), oxy-fluorinated samples with 1 vol% F_2_ (I-CF01/180), and 5 vol% F_2_ (I-CF05/180) as well as a fluorinated CF (I-CF10/180); measurements were done with 10 mm gauge length.

The experimental data were then approximated by a linear equation (*y* = *m·x* + *n*). According to Watanabe et al. [33], the obviously curved downward parts of the Weibull distribution, presented in Figure 9, can be related to easy to break measured weak fibers, which were included in the experimental data.

As shown in Table 4, the maximum tensile strength is significantly different for all conditions in its populations. For 5 vol% (I-CF05/180), the highest tensile strength was determined; also, the Weibull distribution exhibits the highest value for the characteristic breaking strength at ln(−ln(1 − *P*(*σ*)) = 0, as shown in Figure 9. Here, I-CF05/180 obtains the greatest characteristic breaking strength with 8.46 next to a theoretical tensile strength *σ_theoretical_* of 4738.6 MPa (from ln(*σ*)). In addition, all treated samples presented in Figure 9 exhibit an increased characteristic breaking strength compared to the reference sample I-CF00/000. In terms of the high partial values of ln(*σ*) in Figure 9, it can be concluded that an exalted potential of oxy-fluorination affects the tensile strength of carbon fibres. In consideration of the Griffith failure criterion, the improved surface energy assess onto the fiber strength to the square [49], which can be seen by the highest total surface energy for treated samples with 5 vol% (I-CF05/180, Table 3) and the highest tensile strength (Table 4 and Figure 9).

**Figure 10:**
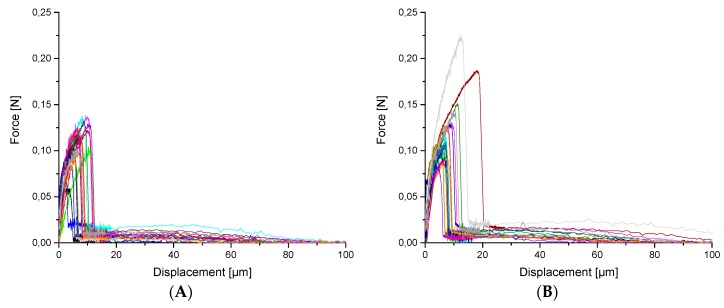

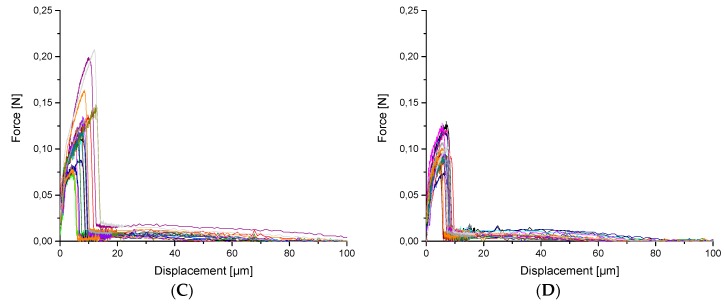
Force-displacement curves for I-CF00/000 (reference) (**A)**, oxy-fluorinated samples with 1 vol% F_2_ (I-CF01/180) (**B)**, and 5 vol% F_2_ (I-CF05/180) (**C)** as well as a fluorinated CF (I-CF10/180) (**D**).

The SFPO test results exhibit no evident difference between calculated shear strengths (Table 5), whereas F_max_ is reached at higher displacements leading to an increase in debonding work W_d_ for the oxy-fluorinated carbon fibres I-CF01/180 and I-CF05/180 (Figure 10).

**Figure 11:**
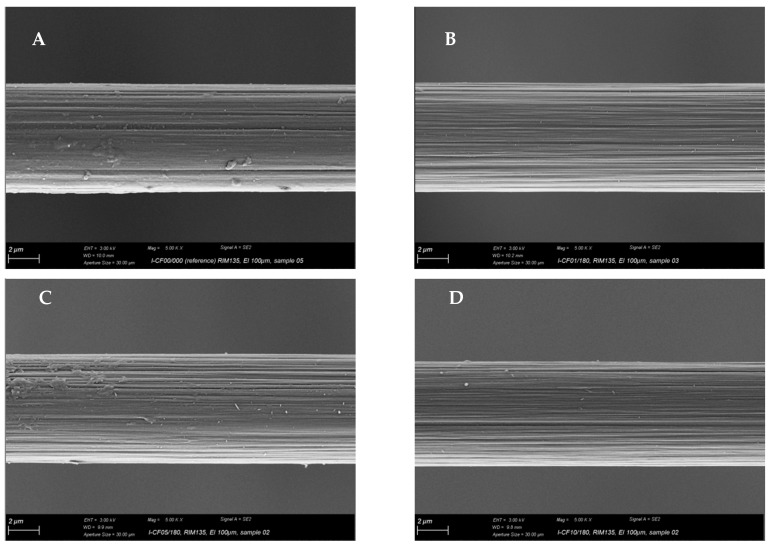
SEM of pulled-out single fibers of I-CF00/000 (reference) (**A**), oxy-fluorinated samples with 1 vol% F_2_ (I-CF01/180) (**B**), and 5 vol% F_2_ (I-CF05/180) (**C**) as well as a fluorinated CF (I-CF10/180) (**D**).

In Figure 11, the surface of the pulled out single fibres is shown. For fibre I-CF00/000 (A), which had a suitable epoxy sizing according to the used matrix, remaining polymer material can be observed on the fibre surface. However, it cannot be clearly stated if this polymer is a sizing or matrix residual. For I-CF01/180, the desizing effect of the oxy-fluorination could be proven by XPS, zeta potential measurements, and tensiometry results. According to this, the surface was additionally functionalised by 5 vol% fluorine in the gas mixture of the process, which resulted in a subsequent increase in surface-bound oxygen (XPS, Table 3 and Figure 6), a decrease in zeta potential (Figure 8), or increase in polar surface energy (Table 3). Also, I-CF05/180 exhibits left over matrix on its surface (Figure 11C), thus confirming enhanced adhesion through an increased work for displacement (Table 5). In addition to the changed surface composition and the described surface morphology influenced by oxy-fluorination (Figure 3), the removal of the weak outer surface layer is caused by this intensive oxidation procedure. Due to this and mechanical interlocking, adhesion is enhanced and the pull-out work is increased.

## 4. Conclusions

The effects of oxy-fluorination treatment on the mechanical and adhesion properties of PAN-based carbon fibers in an epoxy matrix were studied and correlated with the surface characteristics of modified carbon fibers. Due to a commercially available epoxy/polyurethane-sized reference sample, a desizing could be shown by XPS, based on a changed surface composition and an obvious roughening of the surface accompanied by an initial decrease in total and polar surface energy. Increasing the treatment intensity, the best functionalization and tensile strength increase was shown for 5% fluorine in the gas mixture. In this case, sufficient remaining oxygen led to the highest polar surface energy and highest debonding work for SFPO, caused by the high amount of introduced oxygen-containing functional groups at the carbon fiber surface. Moreover, a continuous increase in density and surface-bound fluorine was shown for an increasing fluorine concentration in the gas phase. A production-scale oxy-fluorination plant was used for this research, which makes this type of surface treatment and preparation an attractive option for resource-friendly productions without a wet-chemical treatment of carbon fibers.

## Figures and Tables

**Table 1:** Reactive conditions for oxy-fluorination treatment.

Specimen	F_2_/O_2_ Mixture in N_2_	Conditions
I-CF00/000	No treatment	
I-CF01/180	1 vol%/18 vol%	25 °C/180 s
I-CF03/180	3 vol%/14 vol%	25 °C/180 s
I-CF05/180	5 vol%/10 vol%	25 °C/180 s
I-CF07/180	7 vol%/6 vol%	25 °C/180 s
I-CF10/180	10 vol%/0 vol%	25 °C/180 s

**Table 2:** Density of oxy-fluorinated carbon fibers.

Specimen	I-CF00/000	I-CF01/180	I-CF03/180	I-CF05/180	I-CF07/180	I-CF10/180
Density (g/cm^3^)	1.719 ± 0.034	1.753 ± 0.019	1.768 ± 0.044	1.756 ± 0.024	1.786 ± 0.092	1.803 ± 0.045

**Table 3:** Surface polarity of differently treated carbon fiber samples described by surface free energy (total, polar, and disperse) as well as contact angles for water and diiodomethane (CH_2_I_2_) with variance. For comparison purposes, the elemental ratios [O]:[C] and [F]:[C] were determined by means of XPS.

Sample	σ_total_ (mN·m^−1^)	σ^P^ (mN·m^−1^)	σ^D^ (mN·m^−1^)	θ_water_ (°)	θ_CH2I2_ (°)	[O]:[C]	[F]:[C]
I-CF00/000	60.60 ± 0.42	16.90 ± 0.25	43.71 ± 0.33	49.05 ± 3.30	30,66 ± 6.69	0.238	0.013
I-CF01/180	49.13 ± 0.81	14.27 ± 0.51	34.87 ± 0.64	59.98 ± 5.39	48.33 ± 9.41	0.245	0.047
I-CF03/180	50.68 ± 1.01	14.16 ± 0.58	36.52 ± 0.83	58.89 ± 6.99	45.49 ± 7.40	0.302	0.109
I-CF05/180	52.11 ± 0.79	15.42 ± 0.47	36.68 ± 0.63	56.66 ± 5.57	45.18 ± 7.62	0.319	0.173
I-CF07/180	48.84 ± 0.82	15.66 ± 0.47	33.18 ± 0.68	58.83 ± 5.80	51.83 ± 4.67	0.306	0.238
I-CF10/180	44.62 ± 0.38	15.87 ± 0.22	28.75 ± 0.30	61.98 ± 2.52	59.66 ± 2.98	0.260	0.451

**Table 4:** Mechanical properties of reference and treated specimens, maximum breaking force of single fiber.

Specimen	I-CF00/000	I-CF01/180	I-CF05/180	I-CF10/180
F_max_(mean) (cN)	14.17 ± 2.75	15.08 ± 2.97	15.60 ± 2.87	15.11 ± 2.81
F_max_(median) (cN)	14.33	15.08	15.50	15.39
σ_max_(mean) (MPa)	3538.94	3964.36	4281.16	3975.53

**Table 5:** Adhesion properties of reference and treated specimens, interfacial frictional shear stress as well as different pull-out works.

Specimen	I-CF00/000	I-CF01/180	I-CF05/180	I-CF10/180
τ_app_ (N/mm^2^)	65 ± 10	65 ± 5	68 ± 15	64 ± 6
τ_d_ (N/mm^2^)	66 ± 11	66 ± 7	63 ± 11	62 ± 5
τ_f_ (N/mm^2^)	5.5 ± 3.4	5.5 ± 2.1	4.0 ± 2.0	4.7 ± 2.0
W_d_ (mN/mm)	0.71 ± 0.30	0.97 ± 0.66	0.94 ± 0.48	0.60 ± 0.16
W_pullou_ (mN/mm)	0.70 ± 0.26	0.91 ± 0.46	0.73 ± 0.30	0.61 ± 0.19
W_total_ (mN/mm)	1.42 ± 0.51	1.88 ± 1.09	1.70 ± 0.69	1.21 ± 0.31
l_e_ (µm)	77 ± 17	86 ± 21	76 ± 17	71 ± 11
